# Women, Drug Dependency and Consequences: A Study from a Developing Country

**DOI:** 10.1155/2015/831954

**Published:** 2015-02-23

**Authors:** Mohammad Khajedaluee, Maliheh Dadgarmoghaddam, Majidreza Erfanian, Arash Alipourtabrizi, Majid Khadem-Rezaiyan

**Affiliations:** ^1^School of Medicine, Mashhad University of Medical Sciences, Mashhad, Iran; ^2^Legal Medicine Organization, Mashhad, Iran

## Abstract

*Introduction*. Addiction in women can expose them to malnutrition, high blood pressure, cancer, and some other dangerous diseases like hepatitis, AIDS, or other sexual transmitted diseases. The aim of this study was to assess illegal sexual relations in three groups of women. *Methods*. This is a cross-sectional study that was done on 236 girls and young women aged 16–25 years in 2012 in three groups: vulnerable women who have substance dependency (crimes that had made women incarcerated were considered as vulnerability in this study), invulnerable women who have substance dependency (substance dependent women without a history of incarceration), and a control group (women with no history of substance dependency or being in prison). *Results*. 43.8% of vulnerable women who have substance dependency had extramarital sexual relations; this percentage was 55.8% in invulnerable women who have substance dependency and 1.4% in the control group. Crystal and methamphetamine abuse was higher in addicts who had extramarital sexual relations and alcohol abuse was correlated with unsafe sexual intercourse (*r* = 0.36, *P* = 0.001). There was a statistically significant difference in extramarital sexual relation based on marital status (*P* < 0.001). *Conclusions*. Poverty, drug dependency, divorce, and alcohol consumption make women prone to other high risk behaviors that need more attention.

## 1. Introduction

Physical dependency is described as “an adaptive state that manifests itself as intense physical disturbance when drug use is suspended” [1].

Most people believe that addiction or drug dependency is a male phenomenon and females are less involved in these issues. In the Islamic Republic of Iran, people are surprised by seeing a woman who smokes cigarettes. Although there is no valid census about drug dependency in Iranian women, but the Ministry of Health reports that there is one female drug dependent per 8 males in Iran [2]. The administrators of prisons declare that 50% of female prisoners are in jails regarding drugs and addiction [2].

A study in the United States of America has shown that 7.7% of males in comparison with 5% of females were drug abusers (the sex difference was lower than 3%). In the United States, almost 4.5 million women drink alcohol, 3.5 million abuse prescription drugs, and more than 3 million abused illicit drugs [3].

Addiction in women can expose them to malnutrition, high blood pressure, cancer, and some other dangerous diseases like hepatitis or AIDS. Comparing to males, women may be more prone to AIDS or other sexual transmitted diseases. Besides, they are more susceptible to other gynecological diseases and their complications [4, 5]. Some studies have been done around this subject [6–12]. This study was aimed at evaluating extramarital sexual relations in vulnerable women who have substance dependency (substance dependence in prison), invulnerable women of substance dependence (substance dependence with no history of being in prison), and control groups (women with no history of addiction or being in prison) in Mashhad, a metropolitan in the north east of Iran. The extramarital sexual relation was assessed within the last year.

## 2. Methods

This cross-sectional study was done on 236 girls and young women aged 16–25 years in 2012, in three groups: vulnerable women who have substance dependency (crimes that had made women incarcerated were considered as vulnerability in this study), invulnerable women who have substance dependency (substance dependent women without history of incarceration), and control group (women with no history of dependency or being in prison, individuals who had come to health centers for primary health care).

The sample size was calculated based on the mean difference in two groups based on a previous study, by considering *α* = 0.05 and *β* = 0.2, and 80 persons were calculated for each group.

In a first step the calculated sample size for each group was divided into 2 subgroups: 16–20 and 21–25 years old (to insure including women younger than 20). In the second step, by using a random sampling method the participants were selected from a list of prisoners (Group 1). At the last step substance dependent women who were referred to welfare centers (Group 2) and women who had come to health centers for primary health care (Group 3) were selected based on age matching to the first group.

All interviewers had attended a training class to eliminate the interinterviewer bias. All participants filled the informed consent and then demographic and familial characteristics, alcohol consumption, and main social and sexual behaviors were obtained. All checklists were filed anonymously and data were kept confidential. Results are published anonymously and collectively.

After filling the checklists, the accuracy of answers to questions was checked by reviewing the key questions. All analyses were done using Statistical Package for Social Sciences (SPSS) version 11.5. *P* < 0.05 was selected as statistically significant level.

## 3. Results

Demographic and social characteristics of participants are demonstrated in Table 1. As shown in this table, education, occupation status, personal income, family income, lodging, marital status, and alcohol consumption are statistically different in three groups.

Regarding extramarital sexual relations there was a statistically significant difference between three groups (43.8% in vulnerable group, 55.8% in invulnerable group, and 1.4% in control group; chi^2^ = 52.21, *P* < 0.001). There was no statistically significant difference between vulnerable and invulnerable groups (chi^2^ = 2.29, *P* = 0.15).

**Figure 1:**
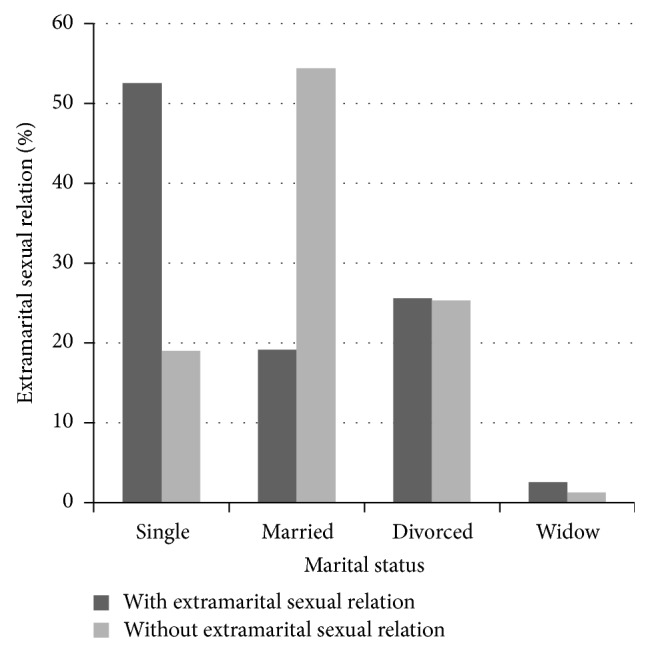
Percentage of extramarital sexual relations in all substance dependent women based on marital status.

The extramarital sexual relation based on marital status in all the substance dependent subjects is shown in Figure 1. The difference was statistically significant (*P* < 0.001).

**Figure 2:**
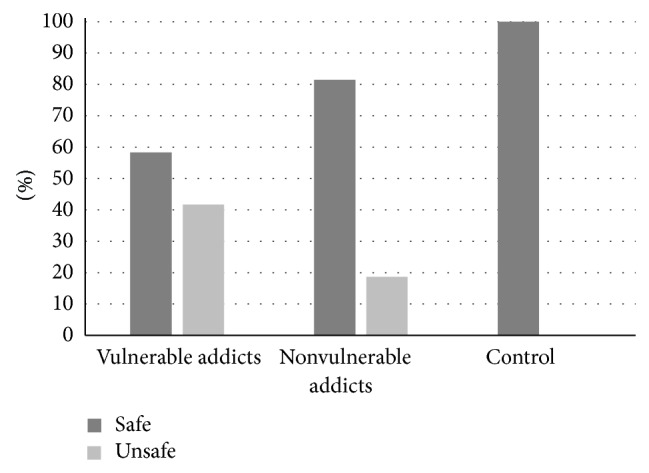
Percentage of condom usage in sexual relations in three groups.

The kind of sexual relation, safe (considered as using condoms) and unsafe (considered as not using condoms), is demonstrated in Figure 2. Among 36 extramarital sexual relations in vulnerable women who have substance dependency, 41.7% were unsafe, and among 43 extramarital sexual relations in invulnerable women who have substance dependency 18.6% were unsafe. In the control group, only one person had an extramarital sexual relation and it was safe. There was a statistically significant difference between vulnerable and invulnerable groups (*P* = 0.03) (Figure 2).

**Figure 3:**
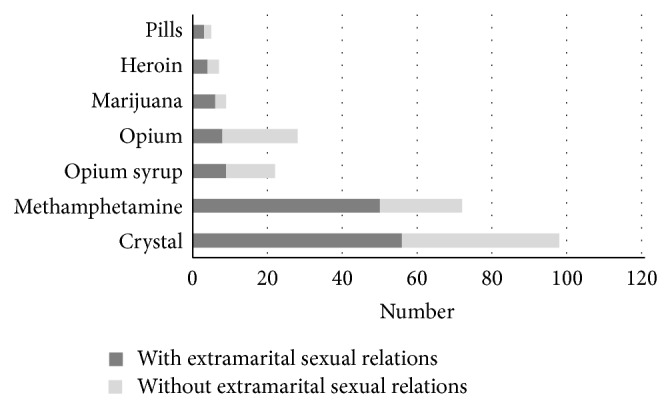
Type of abused drugs based on having or not having an extramarital sexual relation.

As shown in Figure 3, the type of substances which was abused was statistically different in substance dependent participants according to having or not having an extramarital sexual relation (*P* = 0.01).

Alcohol consumption was statistically different in two groups based on safe or unsafe sexual relations (chi^2^ = 9.95, *P* = 0.003). It was 21.2% in safe group and 59% in unsafe group.

## 4. Discussion

43.8% of vulnerable women who have substance dependency had extramarital sexual relations; this percentage was 55.8% in invulnerable women who have substance dependency and 1.4% in the control group. Teets had described the frequency of rape in female drug abusers and found that, from 60 interviewed women, 73% had a history of rape and 45% had been raped more than once. In 35% of participants raping had been happening when the victims are affected by the substances which were abused [6]. In 2010, Perez del Rio et al. studied the relation of drug abuse and showed that in female addicts there is an implicit relation between drug abuse and sexual abuse in childhood or adulthood (37.8%) and in some cases this sexual relation was for earning money (51.4%) [8]. So, it seems that drug abuse affects sexual activities.

In this study, it was shown that crystal and methamphetamine abuse was higher in addicts who had extramarital sexual relations and alcohol consumption was higher in unsafe sexual intercourse group. Although we should notice that crystal and methamphetamine were the most common substances which were abused in all the drug dependent subjects, the type of abused substances was statistically different in two substance dependent women (*P* < 0.01).

Calsyn et al. studied the effects of drug abuse on men who had sexual relation with women. Results of this study showed that condom use in sexual relation was not statistically different when the man had abused drugs in comparison with time they had not used illicit drugs (48.3% versus 49%) [9]. The differences between the results of that study and our study may be due to gender difference.

In one study in Tanzania, Fisher et al. studied safe and unsafe sexual relations in subjects who had drunk alcohol 2 hours before the intercourse comparing to the ones who had not. Results showed that condom usage failure (in males, females, or both) was more than 5 times higher in individuals who had drunk alcohol before the sexual relation especially when the woman had drunk alcohol. Alcohol consumption was higher in some situations: the first sexual relation in women, sexual relations in unknown or with less control era, and people whose parents had abused drugs or alcohol before sexual relations. Surprisingly, condom usage was not related to alcohol consumption but the probability of failure was much higher [10]. In this study we did not assess the condom use failure, so if we did it, the result could be the same, but condom usage was related to alcohol consumption and this difference may be because of different cultural contexts and sexual behaviors in societies.

In this study the extramarital relation was higher in drug dependent women. Ishøy et al. evaluated the extramarital sexual relations in addict women comparing to nonaddict women. Results showed that out of 27 addict women, 14 had a history of extramarital sexual relation. Early abuse of heroin and cocaine was a predictor for prostitution in the future. Depression and somatoform disorders were higher in female drug abusers than the control group. Rape and domestic violence were significant in addicted prostitutes [11].

In this study drug dependent women had a lower educational level but relatively had high personal income and being married was a protective factor for extramarital relation. In another study in Texas, it was demonstrated that addict women had lower education and income than addict men. Higher education and higher age were directly correlated with extramarital sexual relations in men. Extramarital sexual relation was 3 times much higher in single females [13]. The higher rate of extramarital relation in single females was the same as the result of this study.

The results of another study showed that nearly 25.5% of subjects had high risk sexual behaviors in which 47% had not used condoms in the last sexual intercourse. High risk sexual behaviors were higher in heroin addicts, lower ages, and higher income groups [12]. In this study the extramarital relation was higher in higher personal income but this study is a cross-sectional study and we cannot conclude causality from this type of study. One reason may be that this group has an extramarital sexual relation to earn money.

Motazakker et al. studied the high risk behaviors in 384 addicts who were receiving methadone as a treatment. Intravenous method of drug abuse was used by 58 subjects (15.1%), snuff in 235 subjects (61.4%), and oral method in 91 subjects (23.6%). 102 subjects (26.6%) had a history of alcohol consumption for more than 5 years, 88 subjects (22.9%) had tattoos, 6 subjects (1.6%) had needle sharing, 19 subjects (5%) had razor blade sharing, and 62 subjects (16.1%) had a history of extramarital sexual relation. The pattern of drug abuse was as follows: heroin in 127 (33.1%), opium in 194 (50.5%), crystal in 2 (0.5%), crack in 26 (6.8%), and cannabis in 2 (0.5%) individuals [14]. The result of this study emphasizes the role of drug dependency in sexual behaviors. In this study there was a statistically significant difference in extramarital sexual relation based on marital status and being married seems to be a protective factor from extramarital sexual relation.

Based on our study results and some other similar studies [15–18] we can conclude that higher rates of extramarital sexual relations and unsafe sexual intercourse can be the consequence of drug dependency in women. Poverty, the need for money to buy drugs, high rates of divorce in addict women, the need for higher doses to increase sexual arousal, and finally psychological problems with sexual self-esteem form a vicious cycle that can harm this group even more. Addiction, poverty, and family breakdown are among the most important social determinants of health that need more attention, especially in this specific group “substance dependent women.”

## Figures and Tables

**Table 1:** Comparing demographic and social characteristics of three groups.

Factors^*^	Vulnerable (*n* = 80)	Invulnerable (*n* = 80)	Control (*n* = 76)	*P* value
Age	21.10 (3.33)	21.21 (2.39)	20.90 (2.72)	0.75
Education				
Illiterate	12 (15.6)	3 (3.8)	0	<0.001
Primary school	25 (32.5)	18 (22.8)	0	
Secondary school	29 (37.7)	19 (24.1)	12 (15.8)	
Diploma	9 (11.7)	35 (44.3)	30 (39.5)	
Higher diploma	2 (2.6)	4 (5.1)	11 (14.5)	
Bachelor	0	0	23 (30.3)	
Occupation status				
Housekeeper	32 (41)	30 (38)	13 (17.1)	<0.001
Jobless	23 (29.5)	20 (25.3)	10 (13.2)	
Employee	23 (29.5)	29 (36.7)	53 (69.7)	
Income^**^				
Personal	209.11 (163.73)	67.69 (82.85)	147.04 (189.90)	<0.001
Family	340.15 (214.03)	616.66 (177.76)	668.37 (457.19)	<0.001
Lodging				
Center of city	65 (81.3)	55 (68.8)	71 (93.4)	<0.001
Border of city	3 (3.8)	20 (25)	0	
Country	11 (13.8)	4 (5)	1 (1.3)	
Village	1 (1.3)	1 (1.3)	4 (5.3)	
Marital status				
Single	19 (23.8)	38 (47.5)	75 (98.68)	<0.001
Married	28 (35)	31 (38.8)	0	
Divorced	31 (38.8)	9 (11.3)	1 (1.31)	
Widow	2 (2.5)	2 (2.5)	0	
Alcohol consumption	34 (42.5)	13 (16.9)	0	<0.001

^*^Based on the variable, mean (standard deviation) or absolute frequency (relative frequency) is reported.

^**^Based on ten thousand rials.

## References

[1] Wallace R. (2008). *Public Health and Preventive Medicine*.

[2] Safari F. Gender difference in substance abuse and its treatment.

[3] Gordon S. M. (2002). *Research and Professional Training Caron Foundation & Addiction: Gender Issues in Abuse and Treatment*.

[4] European Monitoring Center for Drugs and Drud Addiction (2005). *Differences in Patterns of Drug Use Between Womenn and Men*.

[5] Ghanbarzadeh N., Nadjafi-Semnani M. (2007). A study of HIV and other sexually transmitted infections among female prisoners in Birjand. *Birjand University of Medical Sciences*.

[6] Teets J. M. (1997). The incidence and experience of rape among chemically dependent women. *Journal of Psychoactive Drugs*.

[7] Tuchman E. (2010). Women and addiction: the importance of gender issues in substance abuse research. *Journal of Addictive Diseases*.

[8] Perez del Rio F., Lara F., Gonzalez Gutierrez M. (2010). Sexual abuse, prostitution and emotional dependence in drug addicts. *Revista Espanola de Drogodependencias*.

[9] Calsyn D. A., Baldwin H., Niu X., Crits-Christoph P., Hatch-Maillette M. A. (2011). Sexual risk behavior and sex under the influence: an event analysis of men in substance abuse treatment who have sex with women. *The American Journal on Addictions*.

[10] Fisher J. C., Cook P. A., Kapiga S. H. (2010). Alcohol use before sex and HIV risk: situational characteristics of protected and unprotected encounters among high-risk African women. *Sexually Transmitted Diseases*.

[11] Ishøy T., Ishøy P. L., Olsen L. R. (2005). Street prostitution and drug addiction. *Ugeskrift for Laeger*.

[12] Keshtkar A., Majdzadeh R., Nedjat S. (2012). Characteristics of high-risk sexual behaviors for human immunodeficiency virus infection among Iranian drug abusers. *Journal of Addiction Medicine*.

[13] Medrano M. A., Hatch J. P., Zule W. A., Desmond D. P. (2003). Childhood trauma and adult prostitution behavior in a multiethnic heterosexual drug-using population. *The American Journal of Drug and Alcohol Abuse*.

[14] Motazakker M., Naghadeh M. S., Anosheh M. (2012). The frequency of high-risk behaviors in drug addicted patients referring to methadone treatment centre in Urmia, West-Azerbaijan. *Urmia Medical Journal*.

[15] Bowles M. A., DeHart D., Webb J. R. (2012). Family influences on female offenders' substance use: the role of adverse childhood events among incarcerated women. *Journal of Family Violence*.

[16] James R. (2011). Correlates of sexual self-esteem in a sample of substance-abusing women. *Journal of Psychoactive Drugs*.

[17] Shand F. L., Degenhardt L., Slade T., Nelson E. C. (2011). Sex differences amongst dependent heroin users: histories, clinical characteristics and predictors of other substance dependence. *Addictive Behaviors*.

[18] Izutsu T., Tsutsumi A., Matsumoto T. (2009). Association between sexual risk behaviors and drug and alcohol use among young people with delinquent behaviors. *Nihon Arukoru Yakubutsu Igakkai Zasshi*.

